# Alterations of mucosa-attached microbiome and epithelial cell numbers in the cystic fibrosis small intestine with implications for intestinal disease

**DOI:** 10.1038/s41598-022-10328-3

**Published:** 2022-04-21

**Authors:** Jennifer Kelly, Miran Al-Rammahi, Kristian Daly, Paul K. Flanagan, Arun Urs, Marta C. Cohen, Gabriella di Stefano, Marcel J. C. Bijvelds, David N. Sheppard, Hugo R. de Jonge, Ursula E. Seidler, Soraya P. Shirazi-Beechey

**Affiliations:** 1 Department of Infection Biology and Microbiomes, University of Liverpool, Crown Street, Liverpool, L69 7ZB UK; 2 Department of Physiology, Biochemistry and Pharmacology, College of Veterinary Medicine, University of Al-Qadisiyah, Al Diwaniyah, 58002 Iraq; 3 Arrowe Park University Teaching Hospital NHS Trust, Wirral, CH49 5PE UK; 4 Sheffield Children’s Hospital NHS Trust, Western Bank, Sheffield, S10 2TH UK; 5 Histopathology Department, Sheffield Children’s Hospital NHS Trust, Western Bank, Sheffield, S10 2TH UK; 6 Department of Gastroenterology, Hepatology and Endocrinology, Hannover Medical School, 30625 Hannover, Germany; 7 Department of Gastroenterology and Hepatology, Erasmus MC University Medical Center, PO Box 2040, 3000 CA Rotterdam, The Netherlands; 8 School of Physiology, Pharmacology and Neuroscience, University of Bristol, Bristol, BS8 1TD UK; 9 Present Address: Gastrointestinal and Liver Services, Aintree University Hospital, Lower Lane, Liverpool, Merseyside, L9 7AL UK

**Keywords:** Gastroenterology, Microbial communities, Physiology

## Abstract

Cystic fibrosis (CF) is caused by mutations in the cystic fibrosis transmembrane conductance regulator (*CFTR*) gene. Defective CFTR leads to accumulation of dehydrated viscous mucus within the small intestine, luminal acidification and altered intestinal motility, resulting in blockage. These changes promote gut microbial dysbiosis, adversely influencing the normal proliferation and differentiation of intestinal epithelial cells. Using Illumina 16S rRNA gene sequencing and immunohistochemistry, we assessed changes in mucosa-attached microbiome and epithelial cell profile in the small intestine of CF mice and a CF patient compared to wild-type mice and non-CF humans. We found increased abundance of pro-inflammatory *Escherichia* and depletion of beneficial secondary bile-acid producing bacteria in the ileal mucosa-attached microbiome of CFTR-null mice. The ileal mucosa in a CF patient was dominated by a non-*aeruginosa Pseudomonas* species and lacked numerous beneficial anti-inflammatory and short-chain fatty acid-producing bacteria. In the ileum of both CF mice and a CF patient, the number of absorptive enterocytes, Paneth and glucagon-like peptide 1 and 2 secreting L-type enteroendocrine cells were decreased, whereas stem and goblet cell numbers were increased. These changes in mucosa-attached microbiome and epithelial cell profile suggest that microbiota-host interactions may contribute to intestinal CF disease development with implications for therapy.

## Introduction

Cystic fibrosis (CF) results from mutations in the cystic fibrosis transmembrane conductance regulator (CFTR) gene with the most common causing the deletion of phenylalanine at position 508 of the CFTR protein (F508del-CFTR)^1–3^. CFTR plays important roles in epithelial ion transport and host defence by functioning as an anion channel^4,5^. In the intestine, CFTR mediates chloride, bicarbonate and fluid secretion, with bicarbonate neutralising luminal acidity^6^. Dysfunction of CFTR causes accumulation of dehydrated viscous mucus in the intestine^7^, luminal acidification^8^ and altered motility leading to distal (ileal) intestinal obstructive syndrome (DIOS) in older children and adults and meconium ileus in infants^6,9^. Gastrointestinal disease has significant impact on the lives of individuals living with CF, requiring hospital treatment and possibly surgery. CFTR deficient mice also develop intestinal obstruction upon weaning and do not survive without life-long treatment with osmotic laxatives^10^.

The CF intestine provides an ideal environment for colonisation by opportunistic pathogens influencing composition and diversity of gut microbiota. This impacts the host as the gut microbiome, either directly or indirectly, profoundly influences host nutrition, immune stimulation, and protection against pathogens^11^. Furthermore, by modulating intestinal epithelial cell proliferation and differentiation, gut microbiota maintains intestinal homeostasis ^12,13^. Distinct microbial communities reside in the intestinal lumen and attached to the mucosa, likely due to differing physiological conditions and nutrient availability at each site^14,15^. The proximity of the mucosa-attached microbiome to intestinal epithelial cells promotes host-microbial interactions which have increased potential to influence host health compared with microbes residing in the lumen^16^. Previous studies have shown that the faecal and/or luminal microbiome of CF mice is dysbiotic and less diverse than in wild type mice^17–19^. These studies have also demonstrated that in CF mice there is goblet cell hyperplasia^18^, and retention of mucus and secretory vesicles by goblet and Paneth cells^19^. However, to the best of our knowledge the small intestinal mucosa-attached microbiome of CF mice has not been characterised.

The small intestinal epithelium is lined by a single layer of epithelial cells consisting of absorptive enterocytes and three secretory cell lineages [Paneth, goblet, and enteroendocrine cells (EECs)]^20^. Absorptive enterocytes responsible for transcellular nutrient transport are the most abundant cell type in the small intestine. Goblet and Paneth cells protect the intestinal epithelium by secreting mucus and antimicrobial peptides respectively. EECs play a pivotal role in chemosensing by the intestinal tract^20,21^, and respond to changes in luminal contents by releasing hormones. There are at least sixteen discrete EECs, which produce over twenty different hormones in response to changes in luminal contents that act locally, centrally or in the periphery^22^. These include glucagon like peptides 1 and 2 (GLP-1, GLP-2) secreted by L-type EECs residing more frequently in the distal gut. GLP-1 functions as an incretin hormone, stimulating insulin secretion from pancreatic β-cells, to improve meal-related glycaemia. GLP-2, co-produced with GLP-1, promotes intestinal epithelial cell growth and increased nutrient absorption^23,24^. All four intestinal epithelial cell types differentiate from common pluripotent stem cells located near the base of the crypts in a functionally defined niche. Microbiota residing in the intestinal lumen are key members of the stem cell niche^13^. To maintain homeostasis, the gut epithelium is constantly renewed by division and differentiation of intestinal stem cells. Cell labelling kinetics indicate that absorptive enterocytes, goblet and EECs migrate up the crypt-villus axis turning over every 3–4 days, whereas Paneth cells migrate downward to the crypt base, renewing approximately every 21 days^25^. Notch signalling coordinated with Wnt signalling regulates stem cell renewal and epithelial cell fate^26^. The precise mechanisms by which microbiota may affect Notch or Wnt signalling in the intestine are unknown. However, *Clostridium butyricum* reduces cell proliferation by supressing Wnt/β-catenin signalling^27^ and microbiota-induced, myeloid differentiation primary response 88 (Myd88)-dependent signalling promotes secretory cell fate determination by inhibiting Notch signalling in the intestinal epithelium. These findings connect microbiota activity via innate immune signalling to the Notch pathway, which plays crucial roles in intestinal homeostasis^28^.

*We hypothesised that the abnormal environment of the intestinal lumen in CF promotes microbial dysbiosis, deregulating the proliferation and differentiation of intestinal epithelial cells*.

To test this hypothesis, we investigated the mucosa-attached microbiome and epithelial cell profile in the small intestine of CFTR-null, F508del-mutant and wild-type (WT) mice, a human with CF and in seven non-CF humans. Using Illumina 16S rRNA gene sequencing, we characterised the composition and diversity of the mucosa-attached microbiome and with immunohistochemistry, we analysed the distribution of epithelial cells in ileal tissues. Our results demonstrate dysbiosis within the ileal mucosa-attached microbiota of CF mice and a CF human. They also show a significant increase in the number of stem and goblet cells, and decrease in EECs, Paneth cells and absorptive enterocytes in both CF mice and the CF human, highlighting the role of microbiota-host interactions as a potential basis for some CF-related comorbidities.

## Results

### Altered composition and diversity of the intestinal mucosa-attached microbiome in CFTR-null and F508del-mutant mice

In this study, two sets of small intestinal tissue samples from CFTR-null^29^ and WT littermate mice were obtained from two separate breeding colonies with closely matched conditions, including diet and rearing, from Hannover Medical School (group 1) and Erasmus MC University Medical Center, Rotterdam (group 2). Small intestinal tissues were also obtained from F508del-mutant mice^29^ and littermate WT controls from Hannover Medical School.

**Figure 1:**
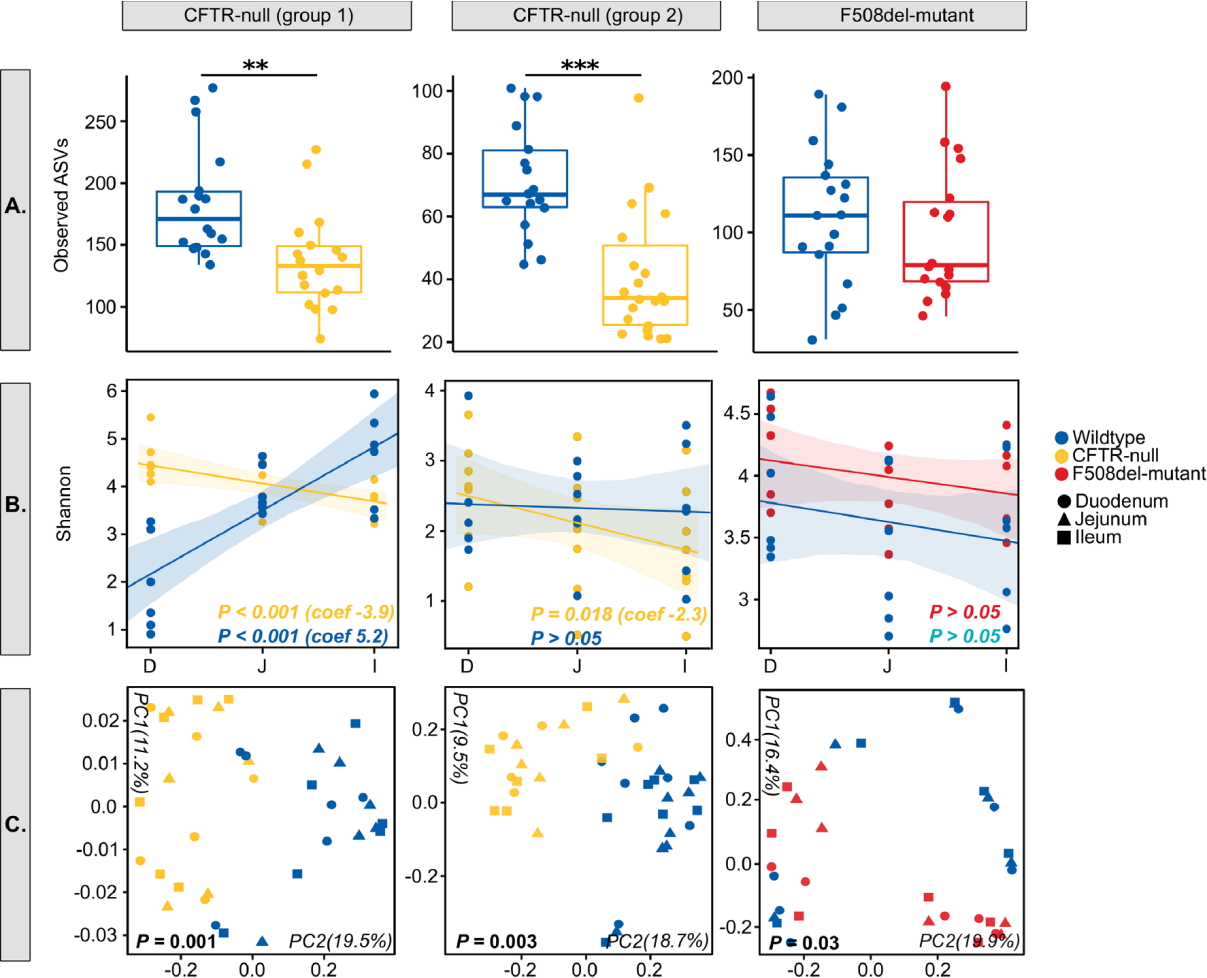
(**A**) Box and whisker plots showing microbial diversity of the mucosa-attached microbiome in CF and WT littermate mice. Boxplots display the median as the midline whilst the perimeters of the box display the 1st and 3rd quantiles of the data. Significance between groups is represented by asterisks; *P < 0.05; **P < 0.01; ***P < 0.001. (**B**) Linear mixed effects regression plots showing the relationship between Shannon diversity and small intestinal region (D; duodenum, J; jejunum, I; ileum). *P*-values and coefficient values are listed for each genotype. (**C**) Principal co-ordinates analysis (PCoA) plots of Jaccard distances for mucosa-attached microbiome samples from CF and WT littermate mice. Colours represent CFTR genotype and shapes represent small intestinal regions. The percentage of variation captured by each PCoA axis is indicated. *P*-values from ANOSIM analysis of Jaccard dissimilarity matrices are listed.

Microbial diversity analysis revealed that in both groups, the mucosa-attached microbiota of CFTR-null mice was significantly less diverse than WT mice (*P* < 0.05; Fig. 1A, Supplementary Table S1). Using regression analysis with small intestinal region (duodenum, jejunum, ileum) as a continuous variable, we tested whether CF mice exhibited an altered pattern of diversity throughout the small intestine compared to WT mice. In WT mice, this analysis revealed that, along the length of the small intestine microbial diversity increased in group 1 but remained constant in group 2 WT mice (Fig. 1B, Supplementary Table S2). By contrast to this, both groups of CFTR-null mice exhibited a significant reduction of microbial diversity along the length of the small intestine (*P* < 0.01; Fig. 1B, Supplementary Table S2). There were no differences in ileal microbial diversity between F508del-mutant and WT mice. (Fig. 1A, Supplementary Table S1). However, analysis of similarities (ANOSIM) testing demonstrated that for both CFTR-null and F508del-mutant mice, composition of the mucosa-attached microbiota was significantly different compared to WT mice (*P* < 0.05; Supplementary Table S3). These findings were confirmed by principal coordinates analysis (PCoA) which showed distinct separation of gut samples from CF and WT mice (Fig. 1C). We also observed a higher degree of dissimilarity between the mucosal microbiomes of CF and WT mice in the jejunum and ileum than the duodenum (Supplementary Table S3).

### The mucosa-attached microbiome of CFTR-null mice is characterised by an increase in *Escherichia* and reduction in secondary bile-acid producing bacterial populations

**Figure 2:**
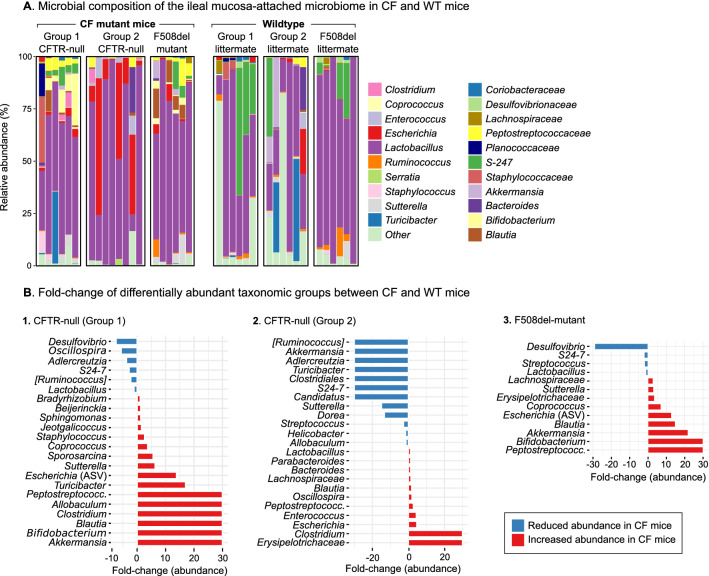
(**A**) Stacked bar plot showing the genus-level microbial composition of the ileal mucosa-attached microbiome in CFTR-null, F508del-mutant and WT littermate mice. Colours represent individual genera. (**B**) Horizontal bar chart showing the fold-change in relative abundance of important taxonomic groups in (1) CFTR-null mice group 1, (2) CFTR-null mice group 2 and (3) F508del-mutant mice compared to their WT littermate controls. Red bars indicate increased abundance in CF compared to WT mice, whilst blue bars indicate reduced abundance in CF compared to WT mice. For the complete list of differentially abundant taxa, see the Supplementary Tables. All taxa exhibiting a fold change above 30 are capped at that value for visualisation purposes.

In the mucosa-attached microbiomes of both groups of CFTR-null mice, abundance of the genus *Escherichia* was increased compared to WT mice, represented by the significant enrichment of an *Escherichia-*classified amplicon sequence variant (ASV) in group 1 (*P* < 0.05, ASV #2) and non-significant enrichment of an *Escherichia*-classified ASV in group 2 (ASV #45) (Fig. 2, Supplementary Tables S4, S5). In group 2 samples we found a higher abundance of the genus *Escherichia* in the jejunum (*P* = 0.09) and ileum (*P* = 0.09) of CFTR-null mice but not in the duodenum (*P* > 0.1; Supplementary Table S5). In group 1 samples, linear regression showed a significant increase of an *Escherichia-*classified ASV longitudinally through the small intestine in CFTR-null mice (*P* = 0.003; ASV #2), but not in WT mice (Supplementary Table S6). Of note, in both groups of CFTR-null mice, the progressive increase of mucosa-attached *Escherichia* along the small intestine culminated in the ileum, where *Escherichia* abundance was highest (Supplementary Tables S4, S5).

Another group of depleted bacteria in CFTR-null mice were those associated with bile acid (BA) metabolism, a process important to host health and severely impaired in CF^30^. In the gut, altered BA levels have been linked to bacterial overgrowth and inflammation^31^. First, in both groups the abundance of the taxonomic group [*Ruminococcus*] was reduced (group 1, *P* = 0.09; group 2, *P* = 0.03; Fig. 2). BLAST analysis revealed that most ASVs in this taxonomic group shared highest sequence identity (96–100%) with strains of *Clostridium scindens* (15 ASVs > 0.1%), a BA-metabolising species^32^ of the recently classified genus *Lachnoclostridium*, as well as *Ruminococcus gnavus* (5 ASVs > 0.1%), *Schaedlerella* (4 ASVs > 0.1%), *Muricomes* (2 ASVs > 0.1%) and *Drancourtella/Sellimona* (1 ASV > 0.1%) (Supplementary Tables S4, S5). Second, the genus *Adlercreutzia* was significantly diminished in CFTR-null compared to WT mice (group 1, *P* = 0.03; group 2, *P* = 0.04; Fig. 2, Supplementary Tables S4 and S5), primarily due to ASVs sharing 100% sequence identity with strains of the closely related genus *Enterorhabdus*; another genus associated with BA metabolism^33^. *Enterorhabdus* belongs to the family *Coriobacteriaceae,* which was also significantly less abundant in group 2 CFTR-null compared to WT mice (*P* = 0.002; Supplementary Tables S4, S5).

Further changes included an increased abundance of the family *Peptostreptococcaceae* (*P* = 0.013) in group 1 CFTR-null (2.89%) compared to WT mice (0.03%), attributable to one ASV with 100% sequence similarity to species within the *Romboutsia* genus (ASV #18, *P* = 0.016) (Fig. 2, Supplementary Table S4). In group 2, the family *Peptostreptococcaceae* (and genus *Romboutsia*) were also more abundant in the ileal mucosa of CFTR-null (2.33%) than WT (0.84%) mice; this difference did not reach statistical significance (Supplementary Table S5). A notable change observed in group 1 CFTR-null mice was a significant increase of the genus *Bifidobacterium* (5.9%) compared to WT mice (0.1%) (*P* = 0.01; Fig. 2, Supplementary Table S4), caused by one ASV with 100% sequence identity to multiple *Bifidobacteria* strains (ASV #4, *P* = 0.04; Supplementary Table S4). Linear regression showed that *Bifidobacterium* abundance increased throughout the small intestine of group 1 CFTR-null mice (*P* = 0.003; Supplementary Table S6) with highest levels observed in the ileum (8.6%). Conversely no changes in levels of *Bifidobacterium* were identified in WT mice, where abundance remained low and stable (Supplementary Table S6).

In summary, using two groups of CF and WT mice raised in different laboratories, we identified many taxonomic groups with consistently changed pattern of abundance between CF and WT mice including *Escherichia,* numerous BA-metabolising bacteria and *Peptostreptococcaceae*. This strengthened the association of these taxa with the CFTR-null genotype and assured the robustness of the data.

### The mucosa-associated microbiome of F508del-mutant mice exhibits a significant degree of dysbiosis

In the mucosa-attached microbiome of F508del-mutant mice, major microbial changes included increased abundance of families *Peptostreptococcaceae* (*P* = 0.001) and *Lachnospiraceae* (*P* = 0.1) compared to WT mice (Fig. 2, Supplementary Table S7). The increase of *Peptostreptococcaceae* was primarily attributable to a *Romboutsia*-classified ASV (ASV #11, *P* = 0.003), with significantly greater abundance in the jejunum (*P* = 0.009) and ileum (*P* = 0.007), while increased *Lachnospiraceae* was due to a higher abundance of genera *Blautia* (*P* = 0.005) and *Coprococcus* (*P* = 0.06) (Fig. 2, Supplementary Table S7). F508del-mutant mice also hosted a significantly greater abundance of *Bifidobacterium* in the jejunum (*P* = 0.0003) and ileum (*P* = 0.005), but not in the duodenum, indicating increased dysbiosis in the ileum of F508del-mutant mice (Fig. 2, Supplementary Table S8). As mentioned earlier, we also observed increased *Bifidobacterium* abundance in CFTR-null mucosal samples (group 1) (Supplementary Table S4).

Linear regression analysis revealed that abundance of an *Enterobacteriaceae*-classified ASV (ASV #2) was significantly associated with small intestinal region, becoming progressively more abundant along the small intestine reaching a relative abundance of 2.8% in the ileum of F508del-mutant mice compared to only 0.12% in WT mice (*P* = 0.039; Supplementary Table S8). BLAST analysis revealed that this ASV shared 100% sequence similarity with multiple strains of *Escherichia* and *Shigella* species including *Escherichia coli*.

Analysis of the mucosa-attached microbiome of F508del-mutant mice showed a reduction of two *Lactobacillus* ASVs compared to WT mice (*P* < 0.1) (ASV #12, #15) (Supplementary Table S7), one of which was also negatively correlated with gut region in F508del-mutant mice but not in WT mice (ASV #15, *P* = 0.06; Supplementary Table S8). In the ileum of F508del-mutant mice the abundance of the genus *Streptococcus* was significantly lower compared to WT mice (*P* = 0.004), as well as several ASVs classified to the family *S24-7* (ASV #8, ASV #17). Notably, a reduction of family *S24-7* was also observed in group 1 CFTR-null mice (Supplementary Table S7).

### Microbial dysbiosis was apparent in the ileal mucosa of a CF patient

**Figure 3:**
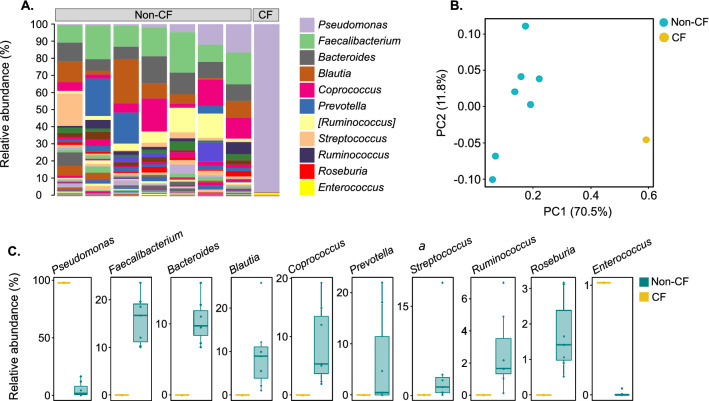
(**A**) Stacked bar plot showing genus-level microbial composition of the ileal mucosa-attached microbiome in a CF and non-CF human sample(s), (**B**) Principal co-ordinates analysis (PCoA) plots from weighted Unifrac distances of the ileal mucosa-attached microbiome from CF and non-CF human samples with colours representing disease status and (**C**) Box and whisker plots showing the relative abundance of the differentially abundant genera in each patient group. Boxplots display the median as the midline whilst the perimeters of the box display the 1st and 3rd quantiles of the data. The percentage of variation captured by each PCoA axis is indicated.

The mucosa-attached microbiome was assessed in ileal tissue of one CF patient previously undergone surgery for DIOS and in ileal biopsies from seven non-CF patients (see Methods). Microbial evenness and diversity were reduced almost tenfold in the CF patient, comprising a total number of 25 ASVs compared to an average 239 ASVs in control samples (Supplementary Table S9B). Dysbiosis in the ileal mucosa-attached microbiome of the CF patient was characterised by the increased abundance of an ASV within the *P. fluorescens* complex. This accounted for 97.6% relative abundance in the CF ileal sample compared to an average 5.3% in control samples (Fig. 3, Supplementary Table S9A). This ASV shared 100% sequence identity to strains of multiple *Pseudomonas* species including *P. antarctica, P. azotoformans, P. costantinii, P. extremorientalis, P. fluorescens, P. poae, P. simiae, P. tolaasii* and *P. trivialis*; all members of the *P. fluorescens* complex^34^. Of note, this ASV did not share 100% sequence similarity with any strains of the dominant CF lung pathogen *P. aeruginosa*. Other ASVs more abundant in the CF microbiome included low abundance genera *Enterococcus* (CF, 1%; non-CF; 0.01%) and *Finegoldia* (CF, 0.2%; non-CF, 0.0004%) (Fig. 3, Supplementary Table S9A).

The major intestinal phyla Firmicutes and Bacteroidetes were both substantially reduced in CF samples, with Firmicutes accounting for only 1.8% relative abundance compared to an average 69.4% in control samples (Fig. 3, Supplementary Table S9A). Bacteroidetes was not identified in the CF ileal dataset (0%) but highly abundant in control samples (18.7%) (Supplementary Table S9). The reduced abundance of Firmicutes and Bacteroidetes in the CF sample was mainly attributable to the depletion of members belonging to genera *Facealibacterium, Blautia, Coprococcus, Ruminococcus*, *Streptococcus, Oscillospira, Roseburia*, *Bacteroides* and *Prevotella*, all of which were highly abundant in control samples (Fig. 3, Supplementary Table S9A). Many of these depleted taxa in the CF ileal mucosa are key commensal gut bacteria associated with host-health benefits and include anti-inflammatory and short-chain fatty acid (SCFA)-producing bacteria.

### Ileal tissues from CFTR-null mice and a CF human exhibit altered intestinal epithelial cell profiles

Using immunohistochemistry, we assessed the number of stem cells and four differentiated cell types: absorptive enterocytes, goblet, Paneth and enteroendocrine cells (EECs), in ileal tissues of CFTR-null mice, WT mice, a CF patient and seven non-CF humans. To identify these cells, we used antibodies to markers of stem cells (Leucine-rich repeat-containing G-protein coupled receptor 5, LRG5)^35^, absorptive enterocytes (brush border membrane, Na^+^/glucose co-transporter 1, SGLT1; cytoplasm, intestinal-fatty acid-binding protein, I-FABP)^36–38^, Paneth cells (α-defensin 5, DEF5)^39^, goblet cells (mucin 2, MUC2)^40^, EECs (chromogranin A, ChA) and L-type EECs (glucagon-like peptide 1 and 2, GLP-1 and GLP-2)^20^.

**Figure 4:**
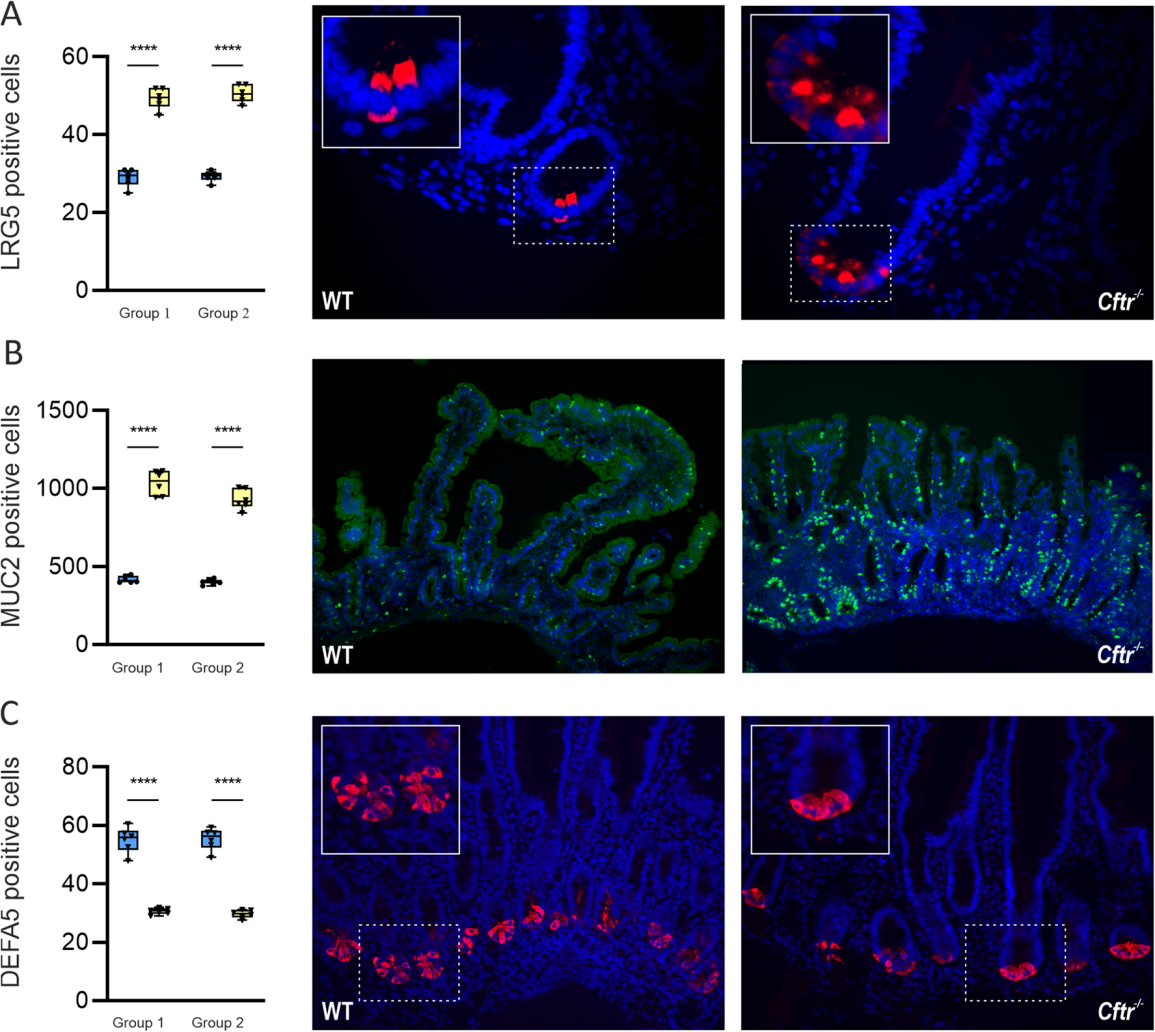
Expression of stem, goblet and Paneth cells in mouse ileal tissue. Quantification of cell numbers and representative images of (**A**) stem cells (leucine-rich repeat- G-protein coupled receptor 5, LRG5) positive cells stained red; (**B**) goblet cells (mucin-2, MUC2) positive cells stained green and (**C**) Paneth cells (defensin 5, DEFA5) positive cells stained red in wild-type (blue square, blue) and CFTR-null (*Cftr*^−/−^) (yellow inverted triangle, yellow) mice from group 1 and group 2. Nuclei are stained blue with 4′,6-diamidino-2-phenylindole (DAPI). Box and whisker plots represent the number of cells counted expressing LRG5, MUC2 and DEFA5 as median ± 95% CI (n = 6 for each mice group). Statistical significance was determined by One-way ANOVA with differences between means identified, using Tukey’s multiple comparison post hoc test; ****P < 0.0001. Images are 400 × (**A**), 100 × (**B**) and 200 × (**C**) magnified. The insets enclosed in white boxes show higher magnification images of the areas enclosed by the dashed lines.

**Figure 5:**
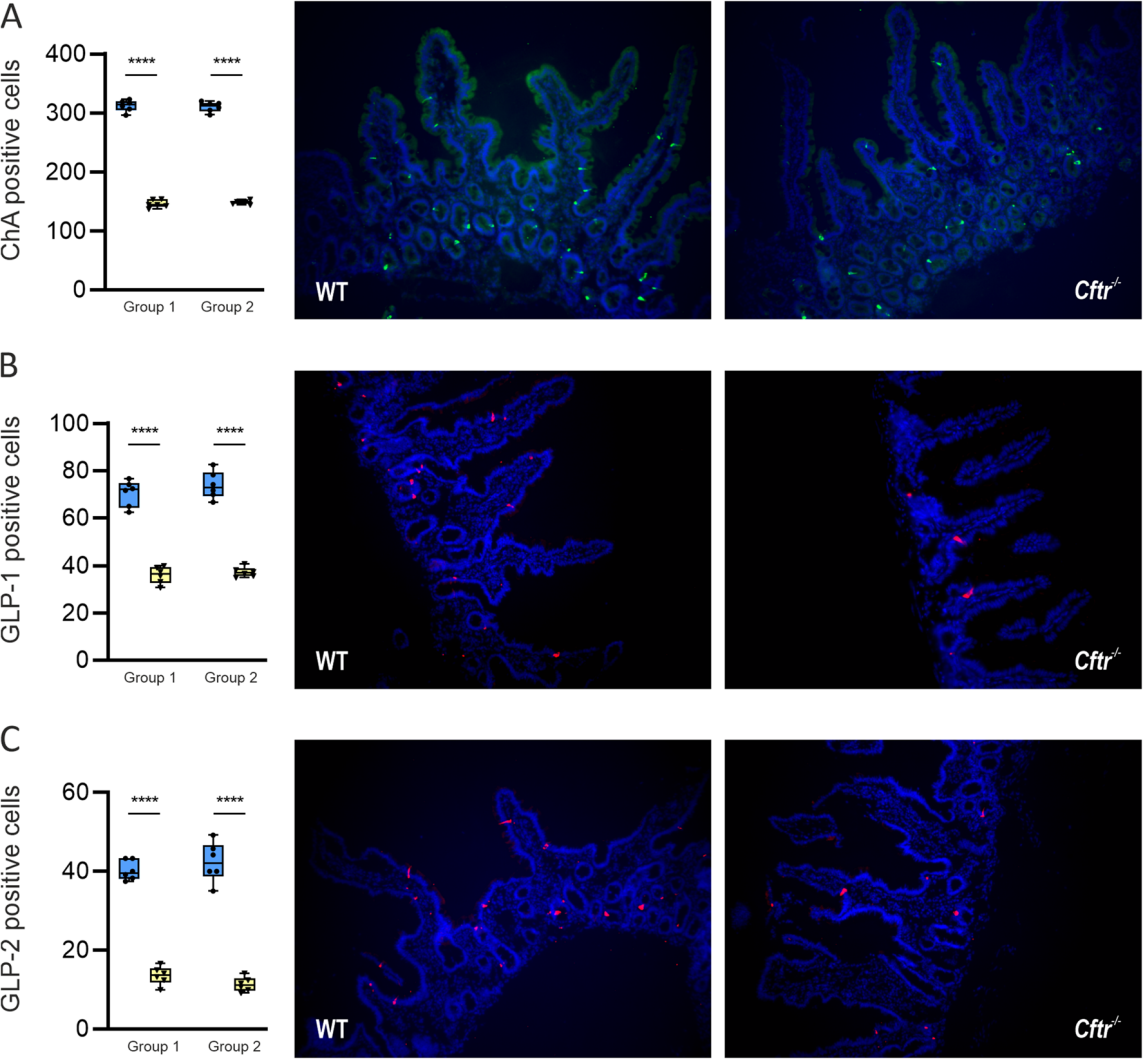
Expression of enteroendocrine cells (EECs) in mouse ileal tissue. Quantification of cell numbers and representative images of (**A**) EECs with ChA positive cells stained green and (**B** & **C**) L-type EECs with GLP-1 and GLP-2 positive cells stained red in wild-type (blue circle, blue) and CFTR-null (*Cftr*^−/−^) (yellow inverted triangle, yellow) mice from group 1 and group 2. Nuclei are stained blue with 4',6-diamidino-2-phenylindole (DAPI). Box and whisker plots represent number of cells counted expressing ChA or GLP-1/2 as median ± 95% CI (n = 6 for each mice group). Statistical significance was determined by One-way ANOVA with differences between means identified using Tukey’s multiple comparison post hoc test; ****P < 0.0001. Images are 200 × magnified.

The total number of different intestinal epithelial cells per high power field of view are given in Supplementary Tables S10 and S11. In ileal tissues of both groups of CFTR-null mice, the number of stem and goblets cells were 1.7- (*P* < 0.001) and 2.3-fold (*P* < 0.01) higher than WT mice (Fig. 4A and B). By contrast, the number of Paneth cells was 1.8-fold (*P* < 0.01) lower (Fig. 4C). The total number of ileal EECs in CFTR-null mice was decreased 2.1-fold (*P* < 0.001) compared to WT mice (Fig. 5A), with the density of ileal L-type EECs containing GLP-1 reduced 3.7-fold (*P* < 0.001) in group 1 mice and 2.9-fold (*P* < 0.01) in group 2 mice (Fig. 5B). In both groups of CFTR-null mice, the number of GLP-2 containing cells was decreased twofold (*P* < 0.001) compared to WT mice (Fig. 5C).

**Figure 6:**
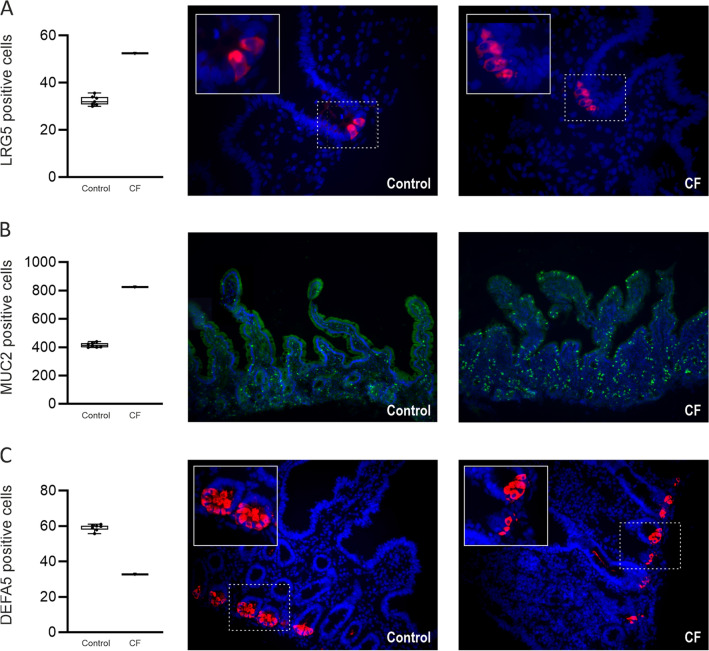
Expression of stem, goblet and Paneth cells in human ileal tissues from one patient with CF and seven non-CF controls. Quantification of cell numbers and representative images of (**A**) stem cells (leucine-rich repeat-containing G-protein coupled receptor 5, LRG5) positive cells stained red; (**B**) goblet cells (mucin-2, MUC2) positive cells stained green and (**C**) Paneth cells (defensin 5, DEFA5) positive cells, stained red. Nuclei are stained blue with 4ʹ, 6-diamidino-2-phenylindole (DAPI). Box and whisker plots represent total number of cells counted expressing LRG5, MUC2 and DEFA5 as median ± 95% CI. The insets enclosed in white boxes show higher magnification of the areas enclosed by the dashed lines. Images are 400 × (**A**), and 100 × (**B)** & 200 × (**C**) magnified.

**Figure 7:**
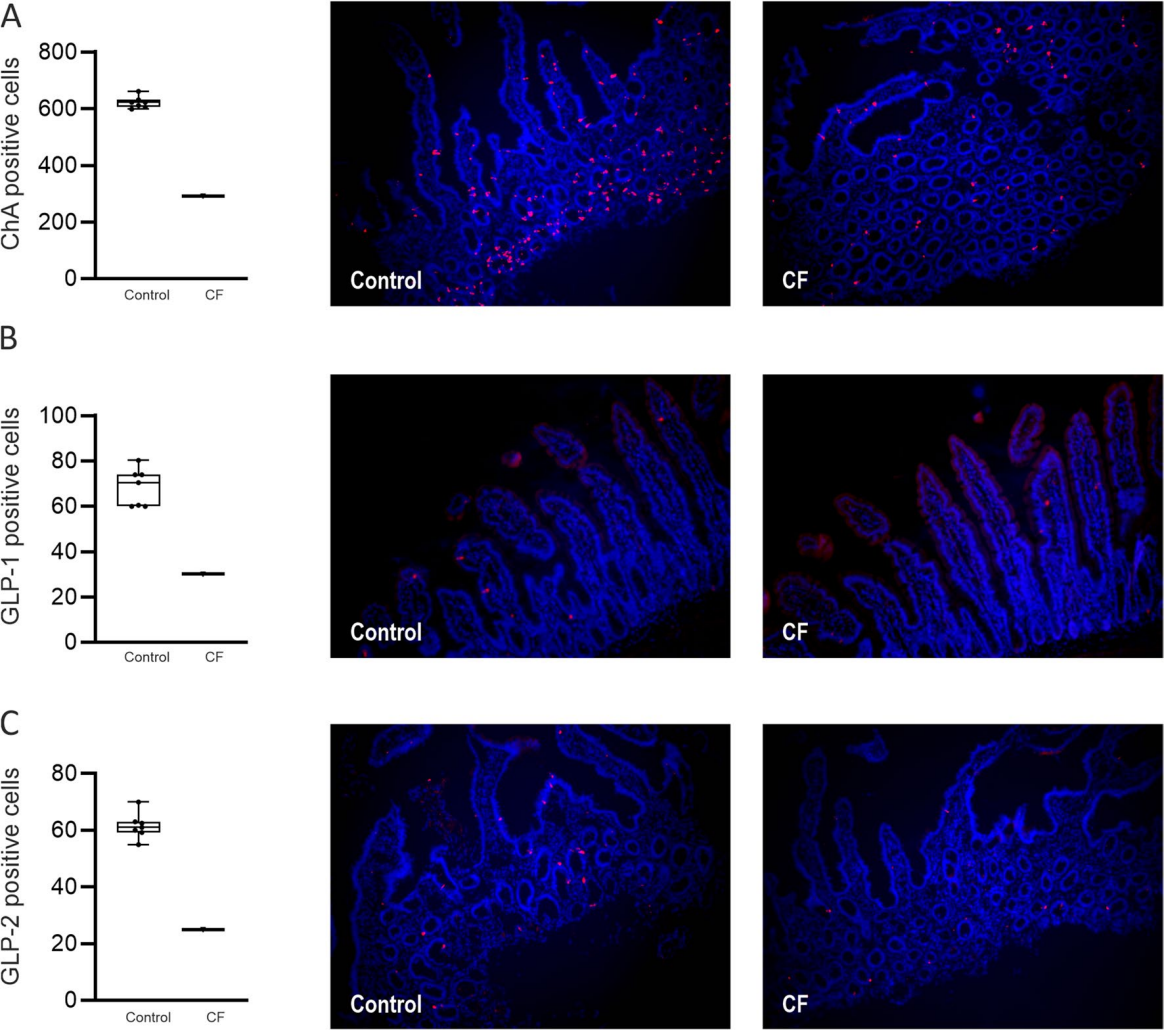
Expression of enteroendocrine cells (EECs) in human ileal tissues from one patient with CF and seven non-CF controls. Quantification of cell numbers and representative images of (**A**) EECs with ChA positive cells and (**B** & **C**) L-type EECs with GLP-1 and GLP-2 positive cells, all stained red. Nuclei are stained blue with 4ʹ, 6-diamidino-2-phenylindole (DAPI). Box and whisker plots represent number of cells counted expressing ChA or GLP-1/2 as median ± 95% CI. Images are 200 × magnified.

Ileal mucosa from a CF patient exhibited a similar intestinal epithelial cell profile to that observed in CF mice. When compared to controls, the number of stem and goblet cells were 1.6- and twofold higher in CF patient ileal mucosa (Figs. 6A and B). By contrast, the number of Paneth cells was reduced 1.8-fold (Fig. 6C), while the density of EECs and L-type EECs possessing GLP-1 and GLP-2 were decreased 2.1-, 2.8- and 2.4-fold, respectively (Figs. 7A-C).

**Figure 8:**
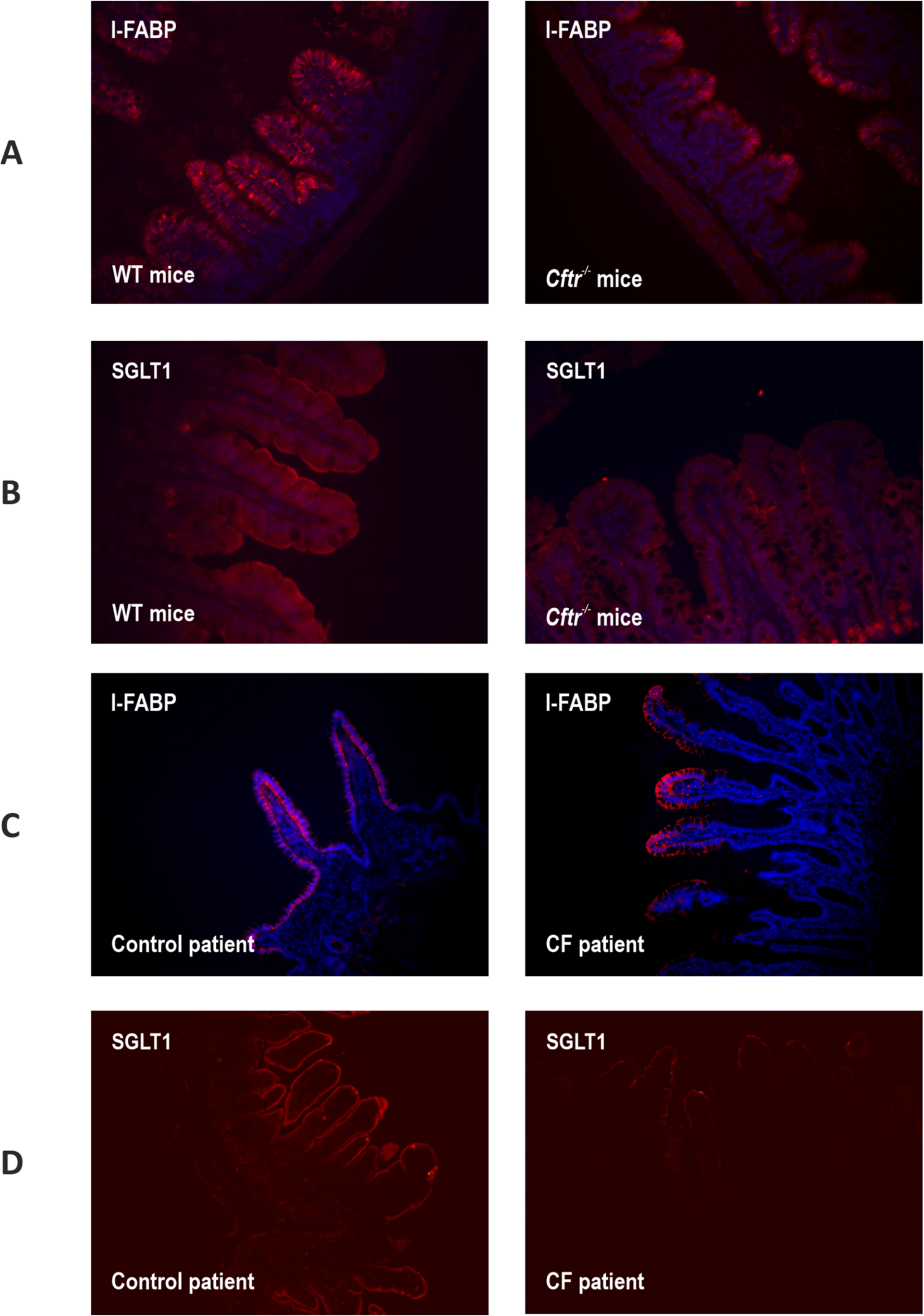
Expression of SGLT1 and I-FABP in absorptive enterocytes of CF and non-CF ileal tissue from mouse and human. Typical immunofluorescent images show cells labelled with I-FABP (**A** & **C**) and SGLT1 (**B** & **D**) in ileal tissues from WT and CFTR-null (*Cftr*^*−/−*^) mice (**A** & **B**) and non-CF control patients and a CF patient (**C** & **D**), all stained red. Nuclei are stained blue with 4ʹ, 6-diamidino-2-phenylindole (DAPI). Images are 200 × (**A**&**D**) and 400 × (**B**&**C**) magnified (n = 6 for each WT and CFTR-null mice group; n = 1 CF and n = 7 control patients).

Consistent with previous results^24,37,38^, in ileal tissues from both WT mice and non-CF human controls, I-FABP labelling was observed in the cytoplasm and SGLT1 labelling on the brush border membrane of absorptive enterocytes along the entire length of crypt-villus axes (Figs. 8A and C). By contrast, in the ileum of CFTR-null mice and a CF patient, I-FABP and SGLT1 labelling were only present on the cells residing at the tips of the villi (Figs. 8B and D). Of note, morphometric analysis demonstrated a 1.4-fold increase in ileal villus height in CF mice compared to WT controls.

## Discussion

In CF, CFTR dysfunction leads to markedly altered gut luminal environment^6^. This distorted milieu promotes dysbiosis of gut microbiota, contributing to gastrointestinal complications such as DIOS, a severe condition that develops in the CF ileum^9^. We used ileal tissue samples to determine changes in the composition and diversity of small intestinal mucosa-attached microbiome in CF mice, and a CF patient compared with WT mice and seven non-CF human controls. Furthermore, in the same ileal tissues (adjacent biopsies/segments) we assessed epithelial cell numbers to identify whether alterations in gut microbiota may influence the profile of small intestinal epithelial cells.

Faecal samples, intestinal contents and colonic biopsies from humans and mice have been used previously to investigate microbial composition and diversity in the CF intestine^17,18,41^. However, the mucosa-attached microbiota in the ileum, the major site of DIOS, has not yet been characterised in CF. It is known that there are significant differences between luminal and mucosa-attached populations^14,15^, while faecal microbiome is not entirely representative of the gut luminal microbiota^42,43^. Mucosa-attached microbiota are in intimate contact with the intestinal epithelium, offering this population greater potential to influence the host than luminal microbiota. They defend against pathogens, modulate immune responses, and contribute to gut-brain communication^44^.

Our results showed that in both CF mice and a CF patient the ileal mucosa-attached microbiota was distinct in composition and had reduced microbial diversity compared to non-CF controls. These data suggest that the altered luminal environment within the CF gut may influence the composition of the normal microbiota. Moreover, our studies of CF mice indicated that the reduced diversity and microbial dysbiosis were more pronounced in the ileum than other regions of the small intestine.

Consistent with previous studies using intestinal contents and faecal samples from CF mice and CF human faecal samples^18,19,45^, our results revealed that both CFTR-null and F508del-mutant mice had increased abundance of the opportunistic pro-inflammatory taxa *Escherichia/Escherichia coli* within the small intestinal mucosa, particularly the ileum. Increased fat^4^ and mucus availability, as well as higher nitrite levels derived from the chronic inflammatory state of the CF intestine^46^ likely promote intestinal *E. coli* expansion. Furthermore, we found that the CF ileal mucosa possessed a lower density of Paneth cells (see below), which mediate specific host immune responses against inflammatory *E. coli*^47^, providing an additional explanation for *E. coli* expansion in the CF gut.

Correlations between inflammatory markers and *E. coli* abundance in CF faecal samples have been suggested^45^. As such, *E. coli* may actively contribute towards the chronic intestinal inflammation exhibited by CF patients and CF mice. Although the mechanisms underlying this effect are unknown, studies demonstrate that within an already inflamed state some commensal *E. coli* strains can elicit further inflammation by stimulating pro-inflammatory cytokines, such as IL-6^48^. We observed higher *E. coli* abundance in the ileum than in either the duodenum or jejunum of CF mice, suggesting that the influence of *E. coli* on host inflammation may be more prominent in the distal small intestine. As inflammation is proposed to play direct and/or indirect roles in DIOS development, increased ileal abundance of *E. coli* may qualify as a contributing factor.

Another gastrointestinal manifestation of CF is impaired BA homeostasis, which results in BA malabsorption and increased faecal BA excretion^30^, contributing to malnutrition and metabolic abnormalities. BAs are mainly absorbed by the sodium-dependent bile acid cotransporter (SLC10A2) expressed on the luminal membrane of absorptive enterocytes of the distal ileum^49,50^. We propose that the reduced number of absorptive ileal enterocytes observed in this study may contribute to diminished BA transport. Moreover, viscous mucus in the lumen of the ileum may prevent BA binding to SLC10A2.

BA homeostasis is controlled by a tightly regulated signalling network with the gut microbiota playing a key role in the biotransformation of primary to secondary BAs^51^. Secondary BAs, absorbed in the distal intestine, not only influence BA homeostasis, but help regulate many intestinal functions, including glucose and lipid metabolism, inflammation and immune homeostasis^52^. We show that several groups of important BA-metabolising bacteria were significantly depleted in the small intestine of CF mice, including the taxa *Clostridium scindens*, *Clostridium hylemonae*^32,53^ and *Enterorhabdus*^33,54^. Our results suggest that microbial metabolism of BAs may be impaired within the CF small intestine, leading to reduced secondary BAs. This idea is supported by studies demonstrating that CF mice and patients exhibit a reduced proportion of secondary BAs within the total BA pool^55,56^. Reduced secondary BA levels in the gut may contribute to impaired BA homeostasis, affecting nutrient metabolism, promoting bacterial overgrowth and intestinal inflammation; all gastrointestinal manifestations of CF.

In agreement with previous studies using CF mouse intestinal contents^19^ and CF patient faecal samples^57^, we observed an increased abundance of *Bifidobacterium* in the ileal mucosa of CF mice. Interestingly, certain *Bifidobacterium* strains promote intestinal mucin production^58^, suggesting a possible contribution to the excess mucus observed in the CF intestine.

We also observed dysbiosis of CF human ileal-attached microbiota, dominated by one species from the *Pseudomonas fluorescens* species complex^34^ and extremely low abundance of many beneficial SCFA-producing genera. The SCFAs acetate, propionate and butyrate are products of microbial fermentation of undigested dietary carbohydrates with beneficial effects on gut health, including maintenance of intestinal barrier integrity, protection against inflammation, and reduced risk of inflammatory bowel disease and colon cancer. Butyrate is also an important energy source for colonic epithelial cells, regulating intestinal tissue homeostasis^59–61^. The observed diminution of beneficial bacterial populations in the CF human ileal-attached microbiota may have been due to the prescribed antibiotic Co-Amoxiclav (see Methods). Consistent with our data showing the striking dominance of *P. fluorescens species* complex in the CF ileum*,* many *Pseudomonas* species including *P. fluorescens* are resistant to Co-Amoxiclav^62,63^. *P. fluorescens* induces proinflammatory responses by intestinal epithelial cells, suggesting that it may contribute to the chronic intestinal inflammation experienced by CF patients^64^. Detection of *P. fluorescens* in CF respiratory samples^34^ suggests that it might originate from the gut as emerging findings indicate that dysbiosis of gut microbiota impacts CF lung disease^65^. There is recent evidence that gut microbiota affects pulmonary immunity through cross talk between gut microbiota and the lungs referred to as gut-lung axis^66,67^, allowing passage of bacterial fragments and metabolites into lymphatic and circulatory systems connecting the gut niche with that of the lung. Moreover, bile acid aspiration linked to gastro-oesophageal reflux is emerging as a major host trigger of chronic bacterial infections and disease progression in CF respiratory diseases, demonstrating interactions between gastrointestinal and lung pathophysiology^68,69^. Deeper understanding of these processes allows the design of suitable pre- and pro-biotics for therapeutic applications.

Although obtaining CF ileal tissue is extremely difficult, a major limitation of this study was use of tissue from only one CF patient. We acknowledge that the CF patient studied may not be representative. However, the microbiota observed in the CF patient was distinct from those found in seven non-CF human controls. Our results provide important insight into gut microbial dysbiosis and deregulation of epithelial cell homeostasis in the CF small intestine. Considering that gastrointestinal manifestations of CF are an understudied priority (https://www.cysticfibrosis.org.uk/news/chewing-the-fat), we urge greater efforts to biobank CF small intestinal tissue.

Besides significant microbial dysbiosis, we found that the ileal epithelial cell population of CF mice was markedly altered. The rapid renewal of intestinal epithelial cells is driven by actively proliferating intestinal stem cells that reside at the base of the crypt in a functionally defined niche^13^. The delicate balance of intestinal stem cells between self-renewal and differentiation controls intestinal epithelial homeostasis. Microbiota residing in the intestinal lumen are key members of the intestinal stem cell niche^13^. In the *Drosophila* gut, there is a complex signalling network between microbiota, immune responses and stem cells^12^. Although these signalling networks are conserved in mammalian species, little is known about the effects of bacteria on renewal of the gut epithelium in mammals. However, stimulation of stem cell activity by invasive bacteria favours the development of hyperproliferative states found in pre-cancerous lesions^70,71^. A similar explanation may account for the enlarged stem cell population we observed in the ileum of CF mice.

We found prominent reductions in the numbers of absorptive enterocytes, EECs and Paneth cells in the ileum of CF mice, whereas goblet cell numbers were notably increased. Increased goblet cell density enhances mucus viscosity, limiting nutrient absorption and contributing to intestinal blockage. Although reduced nutrient absorption in the CF gut also involves pancreatic enzyme deficiency, abnormalities in BA metabolism and altered gut motility, the decreased numbers of absorptive enterocytes and EECs containing GLP-2, which we observed are important additional contributors. The reduction in Paneth cells attenuates the release of antimicrobial peptides that regulate the composition of gut microbiota, escalating gut microbial dysbiosis and promoting inflammation.

The reason for the changes in epithelial cell numbers is incompletely understood, but it has been shown that alterations in transcription factor abundance deregulates cellular differentiation^20,72^. There is compelling evidence that gut microbiota modulates the expression of epithelial cell differentiation factors regulating the profile of gut epithelial cell lineages^20^. In support of this, a recent work demonstrated that in the CF gut, enhanced toll-like receptor-2 signalling, caused by dysbiosis, decreases Notch signalling, enhancing the expression of transcription factors leading to increased goblet cell differentiation, while reducing the expression of Neurogenin 3, the transcription factor responsible for EEC differentiation^73^.

Interestingly, *E. coli,* which we found enriched in the CF ileal mucosa-attached microbiota, is associated with increased intestinal stem cell proliferation^74^ and influences the expression of some intestinal epithelial cell differentiation factors^72^, suggesting its involvement in deregulation of cellular proliferation and differentiation. It is also possible that the *Pseudomonas* species abundantly present in the CF human ileum adversely impacts intestinal proliferation and differentiation.

The decline in ileal L-type EECs secreting GLP-1 slows gut motility and attenuates insulin secretion, whilst the reduction of L-type EECs secreting GLP-2 affects nutrient absorption^20,23,25^. In support of our findings, reduced GLP-1 secretion has been observed in CF patients^75^, leading to the proposition that decreased levels of GLP-1 secretion could affect the development of CF-related diabetes (CFRD)^75^, a condition primarily caused by insulin insufficiency^2^. With better treatments, life expectancy of individuals with CF is rising, but comorbidities such as CFRD also increase with age^2,76^. Little is known about gut hormone-induced insulin secretion in CF, and how defects in the entero-insular axis may contribute to CFRD development. The mechanisms underlying gut hormone-induced insulin secretion in CF, and identification of novel pharmacological therapies (e.g., GLP-1 agonists) require prompt attention. Similarly, the potential role for gut microbiota-host interactions in the development of CF-related comorbidities requires further investigation.

## Methods

### Participant information and human tissue sample collection

Human ileal biopsies from seven non-CF individuals (patients requiring investigative endoscopy for iron-deficiency anaemia) were collected at Sheffield Children's Hospital NHS Trust and Arrowe Park Teaching Hospital, Wirral. Patients were excluded if they suffered from any gastrointestinal disorders or disease. They were age matched as much as possible to the CF patient. Four biopsies were removed from each patient (total of 28 biopsies) and were treated as described below.

CF ileal tissue was obtained in November 2018 from a CF patient at Sheffield Children's Hospital NHS Trust after undergoing closure of ileostomy and colostomy and restoration of intestinal continuity. Eighteen months prior (March 2017), the patient required emergency surgery [subtotal colectomy, ileostomy (30 cm of ileum resected) and sigmoid colostomy] for acute severe/fulminant colitis. Subsequently in May 2018, a pan enteric assessment, including UGI endoscopy, wireless capsule endoscopy, ileoscopy and proctoscopy showed no features of inflammatory bowel disease. On 01/11/2018, following 4 weeks of oral Co-amoxiclav (see Supplementary Table 12 for all medications), the patient’s ileostomy and colostomy were closed, and ileal tissue samples obtained.

Following tissue collection, two biopsies were either wax embedded and sent to Liverpool (see below) or immediately placed in liquid nitrogen and stored at − 80 °C before transfer to Liverpool on dry ice. The frozen samples were stored at − 80 °C until used for determination of mucosa-attached microbiota.

This study was reviewed and awarded ethical permission from the Health Research Authority (HRA) by the Liverpool Central Research Ethics Committee (REC) (REC reference 18/NW/0406, Integrated Research Application System (IRAS) ID IRAS245934) and approved by the University of Liverpool Joint Research Office (JRO) Sponsorship Committee (reference UoL001349). All research was performed in accordance with relevant guidelines and regulations. Signed informed consent was obtained from all subjects and/or the legal guardians prior to tissue collection according to approved study protocol (ID IRAS245934).

### Animals

Group 1 CFTR knockout mice (*Cftr*^tm1Cam^)^77^ and mice homozygous for the F508del mutation (*F508del*^mut/mut^)^78^ and their respective WT (*Cftr* N/N) controls were bred for > 10 generations on a congenic FVB/N background and maintained at Hannover Medical School under special breeding conditions as previously described^79^. *Cftr*^tm1Cam^, *F508del*^*mut/mut*^ and their respective WT littermates were co-housed when feasible, all mice were age matched and used between 10–20 weeks, except where indicated. All animal experiments were approved by the Hannover Medical School and an independent committee assembled by the local authorities (Authorization number: Az. 33.14–42,502-04–14/1549 and Az 33.12–42,502-04–19/3197 for breeding “stressed strains” and Az 33.9–42,502-04–18/2829 for experimental procedures).

Group 2 CFTR knockout (*Cftr*^tm1Cam^; congenic FVB/N) and WT (*Cftr* N/N) mice were obtained from a colony that was maintained by crossing (> 20 generations) of heterozygous offspring at the Erasmus University Medical Center, Rotterdam. Animals were housed in individually ventilated cages in an environmentally controlled room (12 h light/12 h dark cycle, 20–22 °C) at the Erasmus University Medical Center. Experiments were approved by the Independent Committee on Ethical Use of Experimental Animals, Rotterdam, according to national guidelines (141–12-08/10).

Both group 1 and 2 mice received the same drinking solution (40 mM Na_2_SO_4_, 75 mM NaHCO_3_, 10 mM NaCl, 10 mM KCl, 23 g·L^−1^ PEG 4000) to prevent intestinal obstruction in early life^80^ and low fiber diet (crude fiber: 1.5%; protein: 17.8%, fat: 5.2%, carbohydrate: 62.3%; Reference diet 4068.29; AB Diets, Woerden, The Netherlands). Great care was taken to prevent contamination of liquid diets, which could affect gut microbiome secondary to intestinal obstruction. In both groups of CFTR knockout and the F508del-mutant mice, the osmotic laxative included in the drinking water was replaced by normal drinking water 5 days prior to tissue collection. This contrasts with previous studies^18,19^ where laxative treatment was continued until tissue collection, or mice were kept permanently on a liquid diet, Peptamen^6^.

Group 1 animals were euthanised with Isoflurane prior to cervical dislocation, whilst group 2 animals were anaesthetized with Ketamine 120 mg/kg and Xylazine 20 mg/kg (intraperitoneal). For both groups, small intestinal tissue was collected, flushed with ice-cold saline and placed on an ice-cooled glass surface before it was cut open lengthwise. After moisture was removed from the exposed mucosa by gentle blotting with tissue paper, the mucosa was separated from the underlying connective tissue layers by gentle scraping with a glass coverslip. Tissue samples were collected from sections of the duodenum (1–3 cm distal to the pyloric sphincter), jejunum (2 cm section of the middle part of the small intestine), and ileum (2–4 cm proximal to the ileocecal valve). Samples were either fixed and wax embedded or immediately snap-frozen in liquid nitrogen and stored at -80 °C. Wax embedded, and frozen tissues were sent by express post, to Liverpool University either with cool packs or packed in dry ice, respectively.

Each group (CFTR-null, WT and F508del-mutant) supplied by Hannover and Rotterdam consisted of 12 animals. From each mouse two tissue samples were isolated from each intestinal region (duodenum, jejunum, ileum) for i) microbiome analysis and ii) immunohistochemistry. All work with mice complied with Animal Research: Reporting of In Vivo Experiments (ARRIVE) guidelines^81^.

### DNA extraction and sequencing

DNA was extracted from all intestinal tissue samples using the Quick-DNA Fecal/Soil Microbe Miniprep Kit (Zymo Research, Irvine, California, USA) with a 2 × 30 s bead-beating protocol using a Biospec mini bead-beater, resting the sample on ice between bead-beating steps. Purified DNA was quantified using the Quant-iT PicoGreen dsDNA Assay Kit (Life Technologies Ltd, Paisley, UK), with integrity evaluated using agarose gel electrophoresis.

Custom designed primers^82^ were used to amplify the hypervariable V4-region of the 16S rRNA gene from extracted bacterial DNA. Primers comprised the universal forward and reverse bacterial primers 515f. and 806r and the required Illumina flowcell adaptor sequences^82^. The reverse primer also contained a unique 12 base Golay barcode to allow multiplexing of numerous samples. To reduce PCR-associated bias, each sample was amplified in triplicate using a total volume of 25 μl per reaction alongside non-template control reactions. Each reaction mix contained 12.5 µl of Q5 High-Fidelity 2X MasterMix comprised of a thermostable High-Fidelity DNA polymerase, dNTPs and Mg^2+^ (New England Biolabs), 1.25 μl of each primer (10 μM) and 20 ng genomic DNA or water. PCR was carried out using the following parameters: initial denaturation at 98 °C for 30 s, 30 cycles of denaturation at 98 °C for 10 s, annealing at 55 °C for 20 s and elongation at 72 °C for 15 s, followed by a final elongation step at 72 °C for 2 min. For each sample, three replicates were pooled following completion of the PCR reaction. Pooled samples were then combined with 3 μl loading buffer and subjected to 45 min electrophoresis at 100 V in 1 × Tris–Acetate-EDTA (TAE) buffer. Amplified products of the target length were visualised, excised and purified using the QIAquick Gel Extraction kit according to manufacturers’ protocol (Qiagen) before being quantified in triplicate using the Quant-iT PicoGreen dsDNA Assay Kit (Life Technologies Ltd). Quantified samples were combined in equimolar amounts and sequenced on the Illumina MiSeq sequencing platform at the University of Liverpool Centre for Genomic Research (CGR) next-generation sequencing facility. Illumina sequence data have been deposited in the European Nucleotide archive (ENA) under study accession number PRJEB48893.

### Bioinformatic analysis

Raw sequencing reads underwent a strict filtering pipeline to remove low-quality reads. The CGR employed a standard read-filtering pipeline on all sequenced datasets which comprised: i) the removal of Illumina adaptor sequences using CutAdapt^83^ (version 1.2.1); ii) the trimming of low-quality bases using Sickle (https://github.com/najoshi/sickle) (version 1.2), which utilises a sliding window of a defined size to remove read segments which do not have a minimum phred quality value of 20 and iii) the removal of any trimmed reads below 10 bp in length. High-quality paired-end reads were then assembled into overlapping sequences using the assembly software FLASH, based on the following parameters: minimum overlap: 25, maximum overlap: 250, maximum ratio between number of mismatches and overlap length: 0.25^84^. Only assembled sequences above 200 bp in length were retained. Assembled sequences were then filtered for any contaminating phiX sequence carried over from sequencing using BMtagger and the NCBI reference sequence for *Enterobacteria* phage phiX174 (NCBI accession NC 001,422)^85^.

Filtered de-multiplexed reads were analysed using the Quantitative Insights into Microbial Ecology 2 (QIIME2) software package (version qiime2-2021.2, https://qiime2.org)^86^. QIIME2’s “DADA2” plugin was used to resolve reads to high-resolution amplicon sequence variants (ASVs), which represent, as closely as possible, the original biological sequence of the sequenced amplicon^87^. Briefly, DADA2 works by constructing an error model specific to this dataset by training on the whole sequencing run, and then uses this model to correct all sequencing errors in the data and subsequently generate ASVs. The “DADA2” plugin also performs phiX and chimera removal. Following resolution of ASVs, multiple sequence alignment of ASV representative sequences was carried out using MAFFT software, followed by masking of highly variable positions using the QIIME2 alignment plugin. FastTree software was then used to infer unrooted and subsequently rooted maximum-likelihood phylogenetic trees representing the phylogenetic relatedness of ASVs (QIIME2 phylogeny plugin). ASVs were taxonomically classified using a downloaded Naïve-Bayes classifier pre-trained on Greengenes 13_8 99% operational taxonomic units (OTUs) trimmed to include only the 250-bp V4 region bound by the 515F/806R primers utilized in this study (QIIME2 feature-classifier plugin). Following taxonomic classification, ASVs comprising < 10 reads, found in only one sample, or classified as Mitochondria or Chloroplast were removed.

### Microbiome statistical analyses

Microbial diversity and evenness (alpha diversity) was estimated by calculating the following alpha diversity metrics; Shannon’s Diversity Index, Faith’s Phylogenetic Diversity, observed ASVs and Pielou’s Evenness, whilst compositional similarity/dissimilarity between samples (beta diversity) was estimated by generating weighted UniFrac^88^, unweighted UniFrac, Jaccard and Bray–Curtis dissimilarity matrices for all pairwise sample comparisons. Compositional dissimilarity of samples was visualized using principal co-ordinates analysis (PCoA) of beta-diversity distance matrices. To test for significant associations between alpha diversity metrics and categorical metadata groups (i.e., CFTR genotype, Mouse ID, gender), non-parametric Kruskal–Wallis with Benjamini–Hochberg multiple test correction was applied. Spearman’s correlation test was then used to investigate any significant correlations between alpha diversity metrics and small intestinal region (duodenum, jejunum, ileum) by converting intestinal region to numerical metadata. Pairwise comparison of beta diversity distances between categorical metadata groups was analysed using permutational multivariate analysis of variance (ADONIS permanova)^89^ and analysis of similarity (ANOSIM) tests, whilst significant correlations between numerical metadata categories and beta diversity distances were investigated using Mantel tests with 1000 permutations (all tests listed above were performed using QIIME2 diversity plugin). To test for associations between longitudinal changes in alpha and beta diversity throughout the small intestine and CFTR genotype, we performed linear mixed-effects (LME) regression analysis. This accounted for subject-specific variation by using subject/mouse ID as a random effect, whilst allowing identification of longitudinal differences in alpha/beta diversity due to CFTR genotype by using that category as a fixed effect^90^. All analysis described here were carried out using the QIIME2 software package (version qiime2-2021.2, https://qiime2.org)^86^.

Multivariate generalized linear mixed effect modelling was used to identify significant associations between taxonomic relative abundance and CFTR genotype (CFTR-null, WT, F508del) using the software MaAsLin2 (Microbiome Multivariable Associations with Linear Models, https://huttenhower.sph.harvard.edu/maaslin/) with mouse ID and genotype used as random and fixed effects, respectively^91^. *P*-values were controlled for the false discovery rate using the Benjamini–Hochberg procedure. MaAsLin2 analysis was performed using R version 3.6.3 and R studio version 1.3.959 (R Foundation for Statistical Computing, Vienna, Austria).

### Immunohistochemistry

Fresh mouse ileal tissue samples were processed into wax-embedded blocks in Hannover and Rotterdam before transport to Liverpool. They were subsequently sectioned at a thickness of 10 μm, and mounted on polysine-coated slides (Polysine TM, Germany) in preparation for immunohistochemical analysis. Fresh human ileal tissues (CF and non-CF) were placed in formalin immediately after removal and sent to Liverpool where they were wax embedded and sectioned at a thickness of 10 μm. The following antibodies were used for immunohistochemistry: i) chromogranin A: mouse monoclonal anti-chromogranin A antibody (K2H10; 1:300) (Abcam, Cambridge, UK) or an affinity-purified goat anti-ChrA (E-20) antibody (sc-18232; 1:100) (Santa Cruz Biotechnology, Santa Cruz, CA, USA); ii) GLP-1 and GLP-2: affinity-purified goat polyclonal antibodies, GLP-1 (C-17) (sc-7782; 1:100) and GLP-2 (C-20) (1:100; sc- 7781) (Santa Cruz Biotechnology); iii) mucin 2: affinity-purified rabbit polyclonal anti-Muc2 antibody (1:100) (Merck Life Science UK Ltd); iv) defensin 5: rabbit polyclonal anti-alpha 5 defensin antibody (abx319015; 1:200) (Abbexa Ltd, Cambridge Science Park, UK); v) the stem cell marker leucine-rich repeat-containing G-protein coupled receptor 5 (LGR5), also known as G-protein coupled receptor 49 (GPR49); rabbit polyclonal antibody (ARG5526; 1:100) (2BScientific Ltd, Bicester, UK); vi) intestinal FABP (I-FABP): mouse monoclonal antibody (G-5) (sc-376070; 1:100) (Santa Cruz Biotechnology) and vii) SGLT1: AlexaFluor-488 (BS-1128R-A488 raised in rabbit; 1:100) (Bioss Antibodies Inc.,Woburn, MA, USA) and a custom synthesised antibody to mouse SGLT1, raised in rabbits to a synthetic peptide corresponding to amino acids 402–420 (STLFTMDIYTKIRKKASEK)^92^ (1:200) were used. SGLT1 is involved in transport of glucose from the lumen of the intestine into absorptive enterocyte^24^ and I-FABP participates in dietary lipid sensing influencing fat absorption^37^.

Secondary antibodies were used as appropriate, including FITC-conjugated affinity-purified donkey anti-rabbit, anti-mouse IgG and Cy3- conjugated affinity-purified donkey anti-goat IgG (1:500) (Jackson ImmunoResearch Laboratories, West Grove, PA, USA). Immunostaining was visualised using an epifluorescence microscope (Nikon, Kingston upon Thames, UK) and images were captured with a Hamamatsu digital camera (C4742-95). Specificity of immunostaining was determined by omitting the primary antibodies (Supplementary Fig. 1). For the quantification of epithelial cell numbers twelve tissue sections per antibody were used.

All immunohistochemical parameters were tested for normality by the Shapiro–Wilk test. Statistical significance was determined by one-way ANOVA with differences between means identified using Tukey’s multiple comparison post-test. All statistical analyses were performed using Graphpad Prism for Windows Version 9.0 (Graphpad Inc., San Diego, CA, USA). Values were reported as means ± SEM, with *P* ≤ 0.05 considered statistically significant.

## Supplementary Information

Supplementary Figure 1.

Supplementary Tables.
