# Supplementary figures and images for: Alterations of mucosa-attached microbiome and epithelial cell numbers in the cystic fibrosis small intestine with implications for intestinal disease
Source: Sci Rep. 2022 Apr 21;12:6593. doi: 10.1038/s41598-022-10328-3 (PMC9023491; doi:10.1038/s41598-022-10328-3)

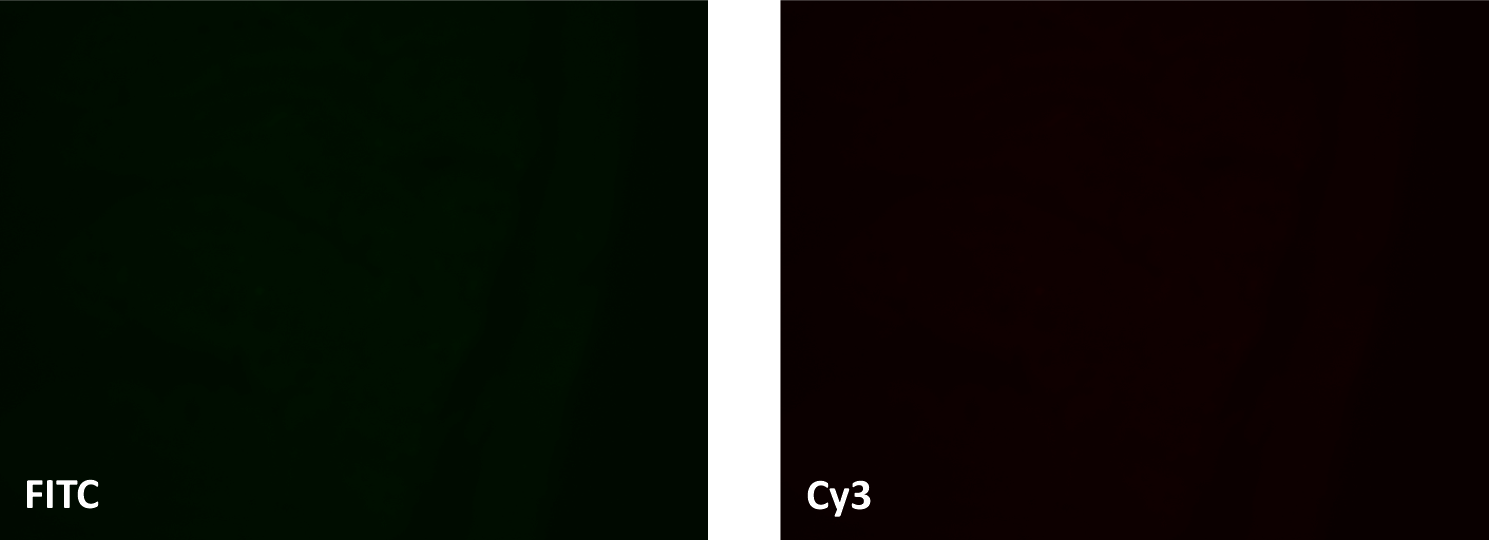

Supplement: Supplementary file 1 — Supplementary Figure 1. [file 41598_2022_10328_MOESM1_ESM.tif]
